# Fostering flow experiences at work: a framework and research agenda for developing flow interventions

**DOI:** 10.3389/fpsyg.2023.1143654

**Published:** 2023-07-07

**Authors:** Karen Bartholomeyczik, Michael T. Knierim, Christof Weinhardt

**Affiliations:** Department of Economics and Management, Karlsruhe Institute of Technology, Karlsruhe, Germany

**Keywords:** flow, work, intervention, intrinsic motivation, job performance, framework

## Abstract

Flow, the holistic experience of intrinsic motivation and effortless attention, is positively associated with job performance, work engagement, and well-being. As many individuals struggle to enter and maintain flow states, interventions that foster flow at work represent valuable catalysts for organizational and individual improvement. Since the literature on work-related flow interventions is still sparse, this article aims to provide a foundation for the systematic development of these interventions. Through a narrative review of the empirical and theoretical field, we develop a comprehensive framework with three dimensions, (1) the intervention aim (entering, boosting, or maintaining flow), (2) the target (context, individual, or group), and (3) the executor (top-down or bottom-up), for systematically classifying flow interventions at work. We complement the framework with guiding questions and concrete starting points for designing novel interventions. In addition, we explain how to build on these dimensions when operationalizing flow as the outcome variable in evaluating intervention effectiveness. By acknowledging individual and situational variability in flow states and the contingent limitations of flow interventions, we offer a broad perspective on the potential for fostering flow at work by using adaptive interventions.

## 1. Introduction

With the average adult with a full-time job spending 8.5 hours at work each weekday (U.S. Bureau of Labor Statistics, 2021), the overall well-being of most employed people is strongly influenced by their job satisfaction (Bowling et al., 2010). Organizations have recognized the influence of well-being on job performance (Wright and Cropanzano, 2000; Ford et al., 2011; Kansky and Diener, 2017) and employee turnover (Richer et al., 2002; Wright and Bonett, 2007). Thus, management increasingly shifts its attention to a more people-centric organization by making efforts to promote individual job satisfaction, work engagement and general mental health (Spreitzer and Porath, 2012; Aarons-Mele, 2018). In this rise of a positive work environment, the concept of flow by Csikszentmihalyi (1975) became popular. Flow is the intrinsically motivating state of optimal experience in which an individual fully concentrates on the current task (Nakamura and Csikszentmihalyi, 2012). Research found that this state occurs more frequently and intensely during work than leisure time (Csikszentmihalyi and LeFevre, 1989; Engeser and Baumann, 2016). However, flow in general is a rare experience that most people struggle with entering intentionally (Ceja and Navarro, 2011; Wilson and Moneta, 2016). Thus, academic literature as well as popular media outlets (e.g., Fisher, 2010; Kotler, 2014; Peifer and Wolters, 2021) call for fostering flow at work to capitalize on its benefits for the individuum (e.g., increased well-being; Bryce and Haworth, 2002), and the organization (e.g., increased performance and creativity; Engeser and Rheinberg, 2008; Zubair and Kamal, 2015).

Despite these repeated calls to increase flow at work, research has only begun to develop and evaluate flow-fostering interventions. For example, in a recent experience sampling study, workers were prompted on five consecutive mornings to write down three goals for the day (Weintraub et al., 2021). This goal-setting nudge increased flow at work, which led to lower levels of stress and enhanced work engagement and performance. However, Weintraub et al. (2021) identified only one other empirical examination of a flow intervention at work by Costantini et al. (2020). This intervention involved a series of behavior change techniques which led to higher absorption at work, a core facet of flow. Based on Weintraub et al.’s (2021) claim of a small empirical field, we conducted a related literature search and identified only two additional intervention studies in the context of flow at work (Drozd et al., 2014; Bartzik et al., 2021). In contrast, there is a larger set of interventions with demonstrated effectiveness for increasing flow in other domains. A recent review from the sport and exercise domain identified 29 studies with interventions (most common: mindfulness interventions, 31%; hypnosis, 17%; imagery techniques, 14%) that were at least modestly successful in increasing flow (Goddard et al., 2021). However, the activities in which flow is experienced during sport and exercise differ substantially from work tasks. While sport and exercise involve high levels of physical activity, every other employee has a (computer-based) desk job (Bitkom Research, 2018). Hence, the interventions from the domain of sport and exercise are hardly applicable to the workplace, as, for example, the use of imagery strategies is difficult in the light of predominantly cognitive job-related tasks. Importantly, 41% of the studies identified by Goddard et al. (2021) also had a single-case design and thus lacked sufficient power to transfer the results to other domains. Nevertheless, the findings from the domain of sport and exercise show that flow is modifiable in principle. In sum, even though earlier evidence indicates that flow at work can be supported, there remains a striking lack of empirical research on flow interventions in this domain.

We attribute the hesitance of the empirical field to address this line of research to three main reasons. First, the empirical field is still debating a common conceptualization of flow (Peifer et al., 2022), especially with regard to its operationalization as continuous or discrete (Abuhamdeh, 2020). However, agreement on how to measure flow in different settings is necessary for the evaluation of intervention effectiveness. Second, flow states at work are highly variable within- and between-persons (Fullagar and Kelloway, 2009; Ceja and Navarro, 2011). Moreover, individual characteristics as well as task type determine the overall likelihood of experiencing flow (Nielsen and Cleal, 2010; Tse et al., 2021), thereby making it difficult to find an intervention that is effective across individuals and jobs. Reducing this complexity in designing flow interventions requires breaking down the end goal into less complex subgoals, thereby providing an anchor for where to start. In fact, Nakamura and Csikszentmihalyi (2012) have already suggested two approaches to fostering flow: targeting the environment or the individual. Both are valuable strategies because flow arises when there is a fit between situational and individual characteristics (Peifer and Wolters, 2021). However, a framework that integrates different goals of interventions with respect to the individual flow state (currently being in flow or not) as well as to distinct environmental or individual targets is still missing. This lack of a framework further impedes the systematic development and evaluation of suitable interventions.

Therefore, we aim to systematize future empirical research on fostering flow at work by providing a comprehensive framework for the scope of flow interventions in this domain. To accomplish this, we first review the concept of flow at work. Based on Walker and Avant’s (2005) concept analysis process, we identify the antecedents, defining attributes, and consequences of flow in a narrative review. We then consolidate the insights from this review into what we call *the sequence of experiencing flow*. We also illustrate the flow concept in a model case and discuss its empirical referents. We then use the sequence of experiencing flow to build our framework, that systematically describes the potential modes of action of flow interventions. To do so, we take into account a person’s current position in the flow sequence, the potential addressees of interventions, and the initiators of interventions at work. Thus, our framework includes three modes of action: (1) aim, (2) target, and (3) executor of the flow intervention. For each mode, we provide exemplary interventions based on the theoretical arguments and/or empirical evidence. We then use these modes to derive guiding questions and a research- and practice-oriented agenda for fostering flow at work. In addition, we discuss the need to consider these modes when evaluating the effectiveness of a flow intervention.

Our article contributes to the flow literature in psychology and management in three major ways. First, by providing three guiding questions based on our framework, we enable researchers to strategically design flow interventions for work. This increases the interventions’ potential taking into account specific goals and situational characteristics. Second, our framework puts forward a concrete research agenda that emphasizes the importance of ensuring that flow antecedents are met. Finally, we provide recommendations for selecting an appropriate flow operationalization to evaluate the effectiveness of an intervention. Thereby, we enable thorough assessments of proposed interventions in terms of increasing the duration, frequency, or intensity of flow depending on the person’s current state. In addition to these implications for researchers, our article also contributes to fostering flow at work in practice. First, we emphasize the importance of targeting the group as a time- and cost-efficient approach to increasing flow, regardless of whether the organization or the employees execute the intervention. Also, we sensitize practitioners to recognize interindividual differences in flow proneness and provide recommendations for integrating them in flow-fostering initiatives.

In the following paragraphs, our article will proceed as follows. First, we will review the literature on the concept of flow and summarize it in the sequence of experiencing flow at work. Second, we will present our framework for flow interventions at work. Finally, we will discuss the framework’s implications for researchers and practitioners, as well as its limitations.

## 2. The sequence of experiencing flow

### 2.1. Antecedents, attributes, and consequences of flow

**Figure 1:**
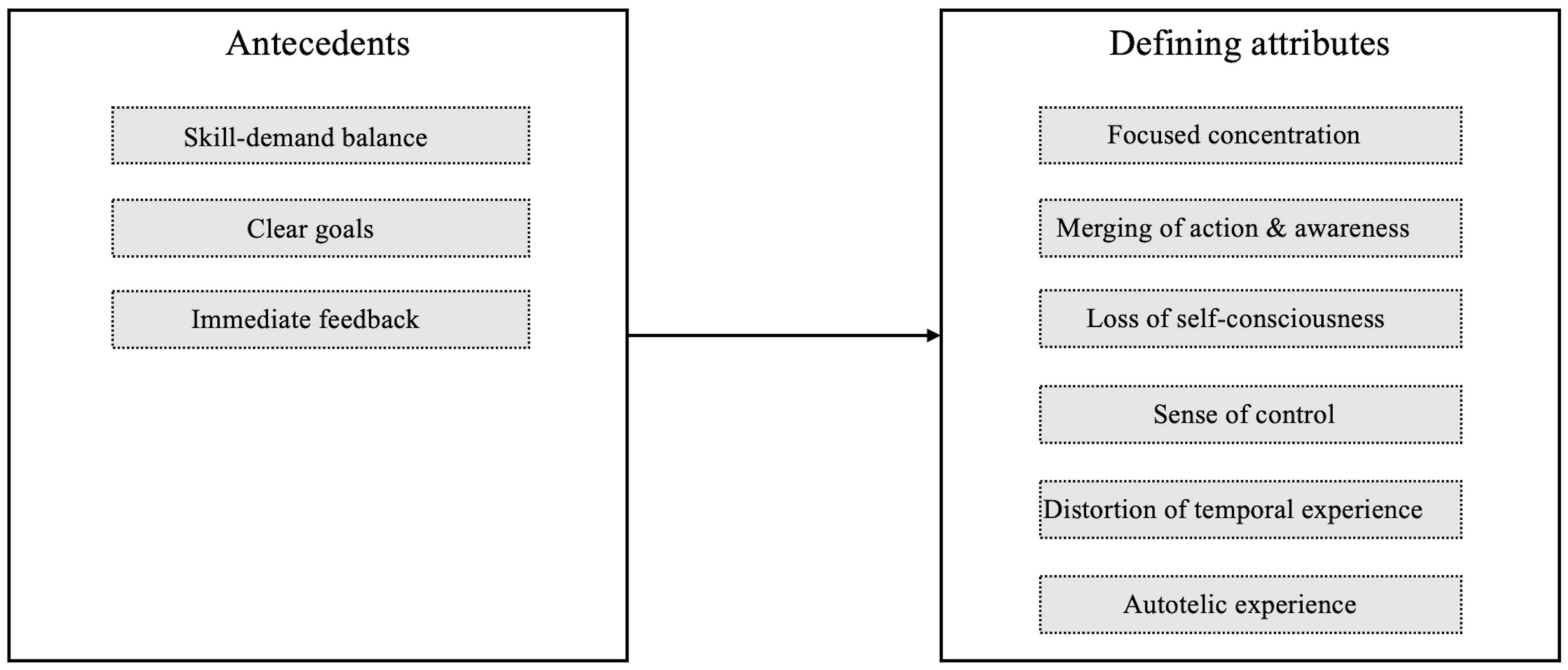
Overview over the flow antecedents and the defining attributes.

Given the ongoing debate about the conceptual modelling and operationalization of flow, for the purposes of this article we follow the argument of Abuhamdeh (2020, p. 9) that “the term flow comes with Csikszentmihalyi’s conceptualization ‘pre-installed’.” According to this original concept, flow has six defining attributes: high concentration, merging of action and awareness, loss of self-consciousness, sense of control, distorted temporal experience, and autotelic (i.e., enjoyable) state. These flow attributes are discriminated from three antecedents, namely clear goals, immediate feedback, and balance of skills and demands (Nakamura and Csikszentmihalyi, 2012; Figure 1).

In addition to these originally defined flow antecedents, flow researchers have identified a number of other variables that are associated with the emergence of flow. In the context of work, Peifer and Wolters’ (2021) distinguish three spheres of these variables, namely social/organizational, job/task-related, and individual variables. Following the original flow concept, we maintain the distinction between the mandatory antecedents (i.e., clear goals, immediate feedback, and skill-demand balance) and these additional influential variables by including the latter as moderators in the sequence of experiencing flow. This is consistent with previous research showing that these variables, e.g., the importance of the task, moderate the relationship between the original antecedents and flow (Engeser and Rheinberg, 2008).

Flow also leads to a series of consequences, e.g., performance or well-being. These are also important for the development of flow interventions, as they will be affected as a result of intervention-induced increases in flow. For the context of work, Peifer and Wolters (2021) aggregated the consequences of flow into the aforementioned three spheres which underlines the potential benefits of fostering flow on an individual, team-related, and organizational level.

**Figure 2:**
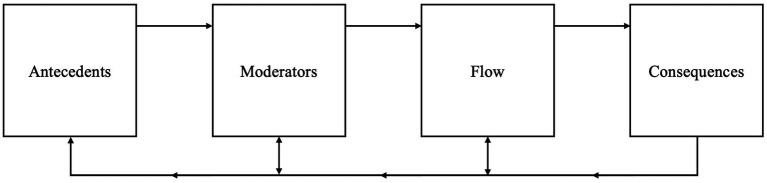
The complete sequence of experiencing flow as a foundation for building a framework for flow interventions at work.

In sum, the flow literature reveals a sequence of experiencing flow with a progression from its antecedents through moderating influences to the defining attributes of the state itself and its associated consequences (Abuhamdeh, 2020; Barthelmäs and Keller, 2021; Peifer et al., 2022; Figure 2). In the next paragraph, we will further illustrate this sequence with a model case.

### 2.2. A model case of flow at work

Hannah works as a data analyst for a large technology company. One of her favorite tasks is programming a data analysis pipeline to predict the success of her client’s advertising campaigns. When she writes the code, she does not have to think twice about how to approach the problem, she just knows what to do. She becomes so absorbed in the task that she stops noticing anything around her. One day, she even missed an important client call because she did not hear the phone. She was too focused to notice the constant ringing. When her colleagues ask her to join them for lunch, she only then realizes that she has not eaten for hours. Sometimes, her partner gets upset on these programming days (she calls them fun days) because she often does not leave in time to make it to their dinner plans.

This model case represents a typical flow experience at work with all its defining attributes. While programming, Hannah is highly concentrated and fully absorbed in the task. Her actions run fluidly without her having to think about it. She feels in full control, experiences time in a distorted way and enjoys the state. She experiences flow in programming because all required antecedents are met. She has clear goals (writing a code that can make accurate predictions), receives immediate feedback (error messages from the programming software), and the task is challenging, but not too difficult for her. Repeatedly experiencing flow in programming allows her to perform well and feel satisfied with her job.

### 2.3. The empirical referents of flow and their relevance for intervention evaluation

Since flow is a subjective experience with strong intraindividual variability (Fullagar and Kelloway, 2009), it is commonly assessed in daily life using self-report measures (Moneta, 2021). Importantly, however, the empirical field has not yet agreed on whether flow is a discrete or continuous construct, i.e., whether there are only two states, flow and nonflow, or whether there is a continuum of flow intensities between these two extremes (Abuhamdeh, 2020; Peifer and Engeser, 2021). Even though Csikszentmihalyi originally defined flow as a discrete state (Abuhamdeh, 2020), most operationalizations of flow are continuous (e.g., Flow Short Scale, Rheinberg et al., 2003). Both conceptualizations bear certain limitations for the evaluation of flow interventions. If flow was defined as discrete (with two states: nonflow and flow), flow interventions could only increase the frequency or total duration of these states. In contrast, if flow was defined as continuous, interventions could increase the intensity (or the duration of flow at a specific intensity level), but the overall duration and frequency of flow could not be assessed (Peifer and Engeser, 2021). This would require the establishment of a specific threshold to distinguish flow from nonflow. Such a distinct boarder not only seems unlikely to exist in work scenarios, but also entails the risk of setting a suboptimal threshold (Abuhamdeh, 2020). Hence, we adopt flow as a yes-or-no continuous phenomenon, i.e., a person experiences the state of nonflow until a threshold is reached, from which the flow state gradually increases on a continuum (Peifer and Engeser, 2021). This is to acknowledge that there are states in which flow is not attainable due to the absence of the antecedents that are, by definition, mandatory for flow to arise (Fullagar and Kelloway, 2009). In our discussion, we will argue how the evaluation of flow interventions in terms of increasing frequency, duration or intensity depends on the current flow state of the person.

## 3. From the sequence of experiencing flow to a framework for interventions

For effective intervention development, it is necessary to circumscribe how an intervention strategy influences the target concept (O’Cathain et al., 2019). With respect to fostering flow, the empirical field has taken two approaches to making this connection. First, it has based interventions on parallelisms between flow attributes (e.g., focused concentration) and the intervention strategy (e.g., mindfulness exercise). However, this approach lacks a specification of how the intervention works, i.e., a specification of its modes of action (Goddard et al., 2021). Second, the empirical field has recently started to translate knowledge about the flow antecedents into interventions (Peifer and Wolters, 2021), for example, by teaching goal-setting strategies to facilitate the availability of clear proximal goals as one major precondition of flow (Weintraub et al., 2021). This approach provides an explanation for the modes of action by arguing that establishing the antecedents of flow should result in a higher likelihood of entering flow. Therefore, it should be favored over the first approach. Following this, we systematically describe the potential modes of action of flow interventions based on the sequence of experiencing flow with distinct antecedents, moderators, and inherent attributes. We do this by clustering the modes of action into a three-dimensional framework, which we present in the following paragraphs.

### 3.1. Aims of flow interventions

**Figure 3:**
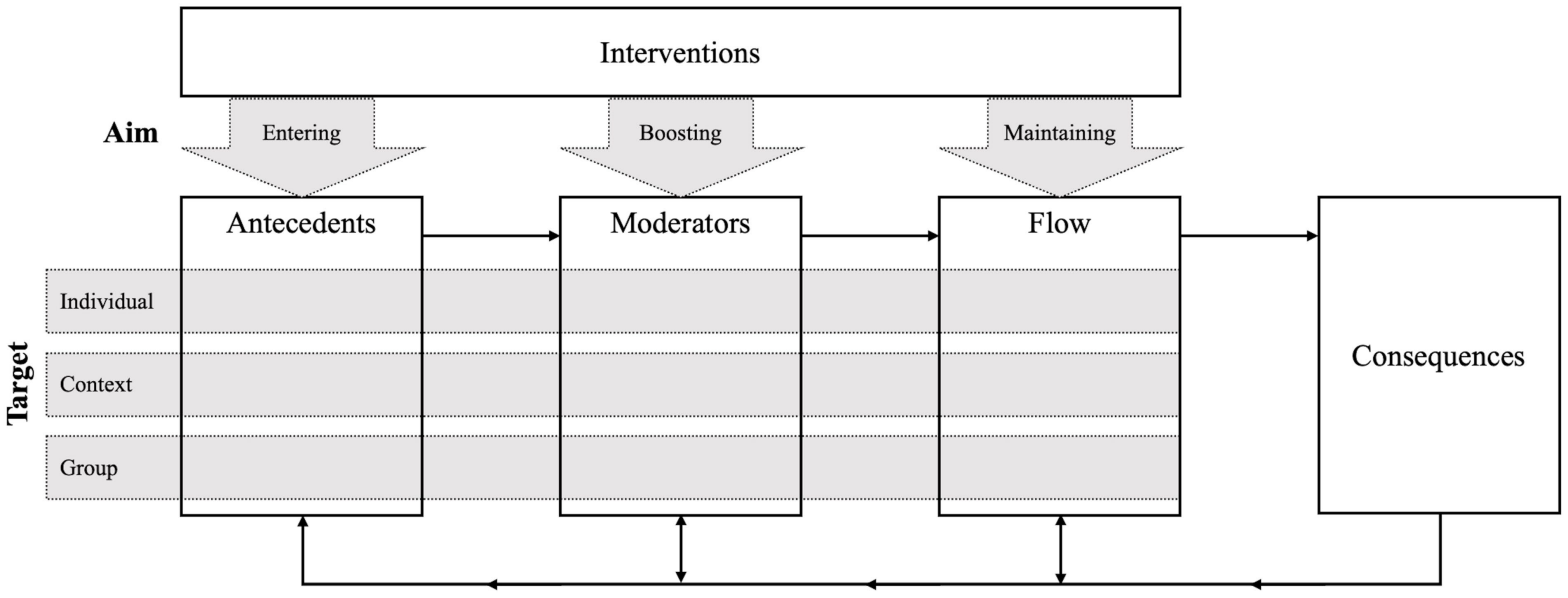
The first and second dimension of the framework for flow interventions at work.

To foster flow, one can have several goals in mind: increasing the frequency of flow experiences, extending the duration of a flow episode, or intensifying the strength of a flow experience regardless of duration and frequency. Thereby, what a specific intervention can accomplish depends on the person’s current flow state. If a person is currently experiencing nonflow, the antecedents are unlikely to be met because their absence diminishes the probability of the occurrence of flow. Conversely, if a person is currently in flow, the antecedents are necessarily given, regardless of the intensity of the state (Fullagar and Kelloway, 2009). Moderators cannot substitute these antecedents or prohibit flow, but they can facilitate entry into flow and influence flow intensity (e.g., Bricteux et al., 2017). Therefore, the components in the sequence of experiencing flow constitute an anchor for the development of flow interventions. We propose that interventions for fostering flow at work can pursue three goals depending on the person’s current flow state and the component on which they focus: (1) entering, (2) boosting, or (3) maintaining flow. Hence, our framework incorporates the intervention aim as the first dimension for classifying flow interventions at work (Figure 3).

#### 3.1.1. Entering flow

Since the antecedents form the basis for the emergence of flow, modulating these variables is necessary to enable crossing the threshold from nonflow to flow. Consequently, these modulations can increase the frequency of flow experiences by increasing the frequency of this transgression. Also, the presence of the antecedents determines the total duration of flow because their withdrawal causes the threshold to be crossed again. Thus, we propose that interventions designed to establish the antecedents pursue the aim of entering flow (Figure 3).

An example of an intervention aimed at entering flow is the aforementioned nudge for setting attainable goals (Weintraub et al., 2021). It aims to establish the flow antecedent of clear proximal goals. Also, previous empirical evidence provides promising starting points for increasing flow by giving feedback, another antecedent in the original flow concept (Peifer et al., 2020; Hohnemann et al., 2022). Thus, establishing structured and regular feedback mechanisms in an organizational context could be effective in enabling people to enter flow at work more often. Finally, presenting tasks with a level of difficulty fitted to the individual’s skill-demand balance is conducive to experiencing flow in experimental settings (Huskey et al., 2018). Thus, management could assign tasks with gradually increasing difficulty depending on individual skills.

#### 3.1.2. Boosting flow

In the sequence of experiencing flow, moderators strengthen the relation between the antecedents and flow. Therefore, modulating them cannot only facilitate the transgression from nonflow to flow provided the antecedents are fulfilled (i.e., influence flow frequency), but also allows to increase the intensity of the subsequent flow states. Hence, we propose that interventions designed for altering the moderators pursue the aim of boosting flow (Figure 3).

As mentioned before, the empirical field for flow interventions at work is still small, but researchers have already suggested different interventions that would conform to this idea. For example, Peifer and Engeser (2021) discuss that providing rewards could foster flow by extrinsically motivating a person to perform an activity that ultimately leads to flow. In addition, Bakker and van Woerkom (2017) argue that job crafting can enhance flow by allowing a person to shape the job to be meaningful and suitable for her. Thereby, job crafting can increase the perceived task importance, a confirmed moderator of the relationship between antecedents and flow (Engeser and Rheinberg, 2008). Additionally, research has identified a set of moderators for the emergence of flow that are related to interindividual differences (e.g., flow metacognitions, Wilson and Moneta, 2016). We will further discuss their potential in the section on targets of flow interventions.

#### 3.1.3. Maintaining flow

Interventions with the above aims, entering and boosting, both apply when a person is not in flow. In contrast, modulating the attributes of the state is only a suitable intervention strategy when the person is currently experiencing flow. In contrast to allowing the transition from nonflow to flow, thereby affecting the frequency and total duration of flow, these interventions can alter the intensity of the current flow experience. Additionally, they can prolong the duration of staying at a specific intensity level, i.e., they can prevent declines on the flow continuum. Hence, we propose that interventions that address flow attributes pursue the aim of maintaining flow (Figure 3).

An interesting idea for such an intervention in an applied context comes from information systems research. Based on real-time classifications of flow using neurophysiological measures, a light could indicate whether a person is currently experiencing flow, thereby preventing interruptions by co-workers (Rissler et al., 2017). In addition to providing diagnostic criteria for flow states, neurophysiological correlates could also constitute intervention targets. For example, Gold and Ciorciari (2019) found that transcranial direct current stimulation increased flow in a computer-based game task. Also, externally evoked changes in the activation of the autonomic nervous system altered the experience of flow (Colzato et al., 2018; Chin and Kales, 2019). However, even though these methods yield promising results in experimental studies, they still need to be translated into interventions that are applicable in the workplace.

### 3.2. Targets of flow interventions

The aforementioned interventions for the three aims differ in their target. For example, while stimulating the autonomic nervous system focuses on the person experiencing flow, blocking interruptions targets the situational context. Therefore, as mentioned above, Nakamura and Csikszentmihalyi (2012) proposed to differentiate flow interventions according to whether they induce changes in the individual or in the environment. The three-spheres framework by Peifer and Wolters (2021) takes on this distinction and further differentiates between external variables related to the job/task or the social/organizational context. Hence, we adopt these earlier differentiations by proposing the intervention target as the second dimension in our framework for classifying flow interventions at work distinguishing between targeting (1) the individual, (2) the group, or (3) the context (Figure 3).

#### 3.2.1. Targeting the individual

We have already touched on empirical evidence that targeting the individual can affect flow (e.g., by teaching goal-setting strategies, Weintraub et al., 2021). Consistent with Person-Environment-Fit Theory (van Vianen, 2018), flow arises when situational and individual variables are aligned (Peifer and Wolters, 2021). Hence, targeting the individual should be especially effective in stimulating flow when individual attributes are modulated to fit the context. For example, nurturing the skills of a person to meet task-related demands may provide the skill-demand balance necessary to evoke flow. This could be done through coaching or training. Importantly, while demographic variables such as gender and socioeconomic status do not strongly predict flow (Isham and Jackson, 2023), individual differences in personality are associated with flow proneness. For example, Ullén et al. (2016) conducted a large-scale twin study and found that dispositional traits explained one-third of the variance in flow proneness. These findings are consistent with Csikszentmihalyi’s (1997) concept of an autotelic personality. Autotelic individuals have a high “need to seek difficulty… and the ability to master it” (Baumann, 2021, p. 237). Empirical research confirms that both high achievement motivation and strong self-regulatory skills moderate the emergence of flow from a skill-demand-balance (e.g., Eisenberger et al., 2005; Engeser and Rheinberg, 2008; Keller and Bless, 2008). Thus, some people may be more responsive to flow interventions or already seek out flow-fostering conditions on their own (Baumann, 2021). Therefore, Wilson and Moneta (2016) argue that training a person to believe in her ability to self-regulate flow helps her to experience flow. However, facilitating long-term counterdispositional behaviors through interventions is more complex than inducing situational changes (Rebele et al., 2021). We will elaborate on this when we discuss the practical implications and limitations of our framework.

#### 3.2.2. Targeting the context

To establish a person-environment fit, one can also target the other side, i.e., the situational context. One of the most prominent models on the influence of the job design is the Job Characteristics Model (JCM; Hackman and Oldham, 1975), which conceptualizes how contextual variables on a job and task level induce psychological states and thereby cause different work-related outcomes. Specifically, it differentiates five job characteristics (skill variety, task identity, task significance, autonomy, and feedback) that lead to certain psychological states (e.g., experienced meaningfulness of work). These psychological states then determine, for example, job satisfaction and performance. Maeran and Cangiano (2013) incorporated flow as one of the psychological states in the JCM and showed that job characteristics, especially feedback and task significance, predict flow at work (see also Engeser and Rheinberg, 2008; Peifer et al., 2020; Hohnemann et al., 2022). Hence, flow interventions that intentionally shape these characteristics should be effective for fostering flow at work. However, to date, no study has evaluated this as a work intervention. Importantly, the feasibility of interventions targeting the context reaches beyond the initiative of the management. The aforementioned job crafting is a perfect example of an intervention that allows a person to change their perceived job significance without requiring the organization’s commitment. Further experimental studies confirm that not only contextual changes at the job and task level, but also configurations of the setting, such as working in a virtual reality environment or in a closed compared to an open office, can increase flow (Ruvimova et al., 2020; Schutte, 2020). The social and organizational context also plays an important role in the occurrence of flow (Peifer and Wolters, 2021). For example, since focused concentration is one of the core attributes of flow, blocking interruptions from coworkers could be an effective strategy for fostering flow. However, current presence of others does not necessarily interfere with flow (Walker, 2021). In the next paragraph, we will elaborate on how promoting interactive teamwork by targeting the group rather than the individual can further enhance flow at work.

#### 3.2.3. Targeting the group

Work by its very nature involves social situations, i.e., people are constantly interacting with others at work. While solitary flow is characterized by the absence of interruptions by others, social flow (also called group or team flow) is a collective, interactive state (van den Hout et al., 2016; Walker, 2021) that “occurs *because* of the presence of others” (Walker, 2021, p. 264). The emergence of social flow largely depends on situational characteristics (Knierim et al., 2019; Walker, 2021) and can therefore be targeted independently of individual traits. Previous research suggests that flow is not only more intense in interactive compared to solitary tasks (Magyaródi and Oláh, 2017) but also perceived as more enjoyable (Walker, 2010). Hence, facilitating a collective flow experience for *all* group members may be particularly fruitful for promoting work performance, creativity, and intrinsic motivation (Walker, 2010; van den Hout et al., 2016). Social flow builds on the antecedents and attributes of individual flow, but comes with additional prerequisites, such as perceived psychological safety, or strong identification with the common goal in the group (van den Hout et al., 2016; Walker, 2021). Thus, targeting the group goes beyond the aforementioned individual or contextual targets for fostering flow. Group-targeting flow interventions should first generally increase opportunities for social flow by assigning shared tasks with high interdependence among group members (Walker, 2010, 2021; Aubé et al., 2014). In addition, interventions could apply team goal-setting strategies to increase commitment to common goals (Aubé et al., 2014), or facilitate role clarification to enable effective task division (Shuffler et al., 2011). Basing rewards on team rather than individual performance or rewarding strong social networks among employees may also provide strategies for reinforcing social flow (May et al., 2004; Aubé et al., 2014; Newman et al., 2017; Walker, 2021).

In sum, each component of the sequence of experiencing flow bears individual, contextual and group-related targets. Thus, interventions with either target can be applied for each aim, i.e., for entering, boosting, or maintaining flow. Of note, all targets should be considered as having equivalent weight. First, there is a necessary fit of the context and the individual as one precondition of flow. Thus, changes on either side (context or individual) can establish this fit because both can be adjusted to the given state of the other. Besides that, targeting the group further fosters flow by facilitating collective flow experiences.

### 3.3. Executors of flow interventions

**Figure 4:**
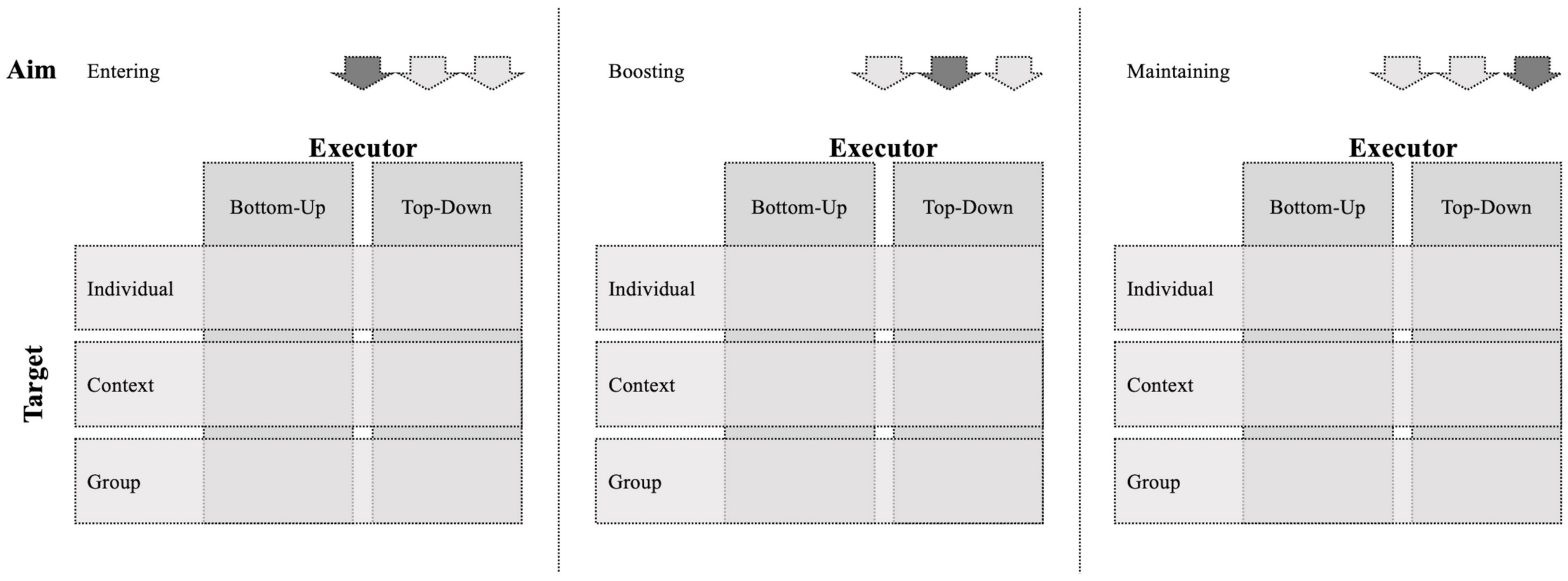
The second and third dimension of the framework for flow interventions at work.

Given the hierarchical dependencies common in the workplace, one may argue that often only management is entitled to employ intervention changes. This assignment of the worker to the role of a passive recipient, rather than an active agent, greatly restricts the applicability of flow interventions at work. However, Bakker and van Woerkom (2017) have proposed the Self-Determination Theory of Flow, arguing that a person can also shape the job and tasks on their own responsibility, thereby allowing them to proactively foster their flow. Hence, in the following paragraphs we introduce a third dimension for classifying flow interventions at work, the intervention executor (Figure 4). In doing so, we build on a review that distinguishes between bottom-up and top-down interventions to increase work engagement (Knight et al., 2019). Proactive initiative of the respective individual characterizes bottom-up interventions, whereas the management applies top-down interventions in a larger organizational context (Hornung et al., 2010).

#### 3.3.1. Top-down execution

Traditionally, interventions at work have been initiated in a top-down manner and were mostly oriented towards increases in work performance (Hornung et al., 2010). However, with the current rise of a people-centric organization, managers have increasingly sought to enable workplaces that also promote individual well-being. With regard to targeting the context, management could, for example, instantiate the aforementioned job characteristics that have been found to predict flow, such as feedback or task significance (Engeser and Rheinberg, 2008; Maeran and Cangiano, 2013; Peifer et al., 2020; Hohnemann et al., 2022). Also, de Sampaio Barros et al. (2018) propose that enhancing autonomy increases the subjective importance of the task. Thus, by allowing the person to work on a task of her own choice, the organization could grant a higher level of autonomy, thereby fostering flow. Besides, organizations could provide quiet workspaces that prevent interruptions, thereby allowing the person to fully focus on their tasks (Rissler et al., 2017). In addition to employing such contextual changes, organizations could target the individual directly, for example, by offering coaching or teaching. This enables enhancements in the skills of the respective person, thereby allowing them to meet the job demands. By encouraging teamwork and fostering information exchange in interdependent tasks (i.e., targeting the group), organizations can also facilitate the emergence of social flow. In sum, because top-down interventions can be directed at the antecedents (e.g., by assigning challenging but achievable tasks), moderators (e.g., by providing performance-based rewards; Peifer and Engeser, 2021), or at the flow state itself (e.g., by blocking interruptions through adaptive information systems; Rissler et al., 2017), they can be implemented not only for each target (i.e., individual, group, or context), but also for each aim (i.e., entering, boosting or maintaining) of fostering flow at work.

#### 3.3.2. Bottom-up execution

Analogous to these top-down approaches, we have already discussed examples of bottom-up interventions with contextual, individual, and group targets. For targeting the context, the strategy of strengths use is a promising tool for fostering flow that transfers the agency to the individual (van Woerkom et al., 2016). It involves the identification of tasks within the given scope of the job that allow the application of individual strengths. In doing so, it induces a subjectively experienced skill-demand balance, which is a major precondition of flow. Moreover, these strengths use interventions can also target the individual by supporting the person identify their individual strengths in the first place. Indeed, strengths use has been found to be associated with flow at work (Liu et al., 2021). To date, strengths use interventions have only been evaluated with regard to flow-related concepts, such as work engagement (Donaldson et al., 2019; Bakker and van Wingerden, 2020). Work engagement is a positive state of vigor, dedication, and absorption in the task (Schaufeli et al., 2006). Even though it closely resembles flow, it is a more perseverant state that lacks the peak experience characteristic of flow (Hallberg and Schaufeli, 2006; Schaufeli et al., 2006). Hence, while strengths use strategies appear to be promising tools for fostering flow, they still need to be empirically evaluated for accomplishing this goal. As top-down interventions, bottom-up approaches can be implemented for all aims, but there is a peculiarity with regard to the aim of maintaining flow. As discussed, interventions with this aim apply when the person is currently experiencing flow. Since flow is characterized by complete absorption in the task (Nakamura and Csikszentmihalyi, 2012), any conscious activity unrelated to that task would immediately disrupt the person’s flow. Behavioral and neurophysiological evidence, however, shows that highly automated, habitual tasks can be performed without distraction from the actual task (Lisman and Sternberg, 2013). Hence, bottom-up interventions can be used for the aim of maintaining flow, but only if the respective person has learned to execute them before entering flow. For example, they could learn to adaptively increase the height of their standing desk. Thereby, they could modulate their neurophysiological activation (Labonté-LeMoyne et al., 2020) to maintain the optimal physiological activation for flow (Peifer and Tan, 2021). However, this would only be an efficient strategy for maintaining flow if they had learned to do this without directing their attention to it.

In sum, we believe that it is important to investigate both bottom-up and top-down interventions because this empowers individuals and organizations alike. As described above, both types of executors can apply interventions with regard to each aim and target, i.e., across the other two dimensions of our framework.

## 4. Discussion

In this article, we proposed a three-dimensional framework for fostering flow at work based on a narrative review of research on the sequence of experiencing flow. Our framework classifies flow interventions by distinguishing between the dimensions of the intervention aim (entering, boosting, or maintaining flow), target (context, individual, or group), and executor (top-down or bottom-up). We sourced our proposals by showing how primary empirical studies and conceptual proposals for intervention strategies fit into the framework’s structure. In the following paragraphs, we further discuss how our framework contributes to research and practice by providing concrete recommendations for its theoretical and practical application.

### 4.1. Theoretical implications

Our article contributes to the literature on flow at work in three major ways. As a first contribution, our framework provides a structure for developing flow interventions by equipping researchers with three guiding questions (Table 1). First, researchers should answer what the goal of the intervention is, i.e., whether the intervention aims to support people who are not experiencing flow at all in their current work situation at all, to enable deeper or more frequent flow experiences, or to help sustain flow. This is consistent with the idea of programme theory, i.e., “developing a causal modal linking programme inputs and activities to a chain of intended or observed outcomes, and then using this model to guide the evaluation “(Rogers, 2008, p. 30). Clearly identifying the aim of an intervention and how it can achieve that aim in a particular setting is a key step in intervention development (O’Cathain et al., 2019). Second, researchers should consider what kinds of changes are possible in the workplace they are focusing on, specifically whether it is possible to modify external factors related to the work setting or the job itself. Finally, researchers need to answer whether they want to develop a strategy for implementation by the management or by the individual worker. It is important to consider not only the likelihood of change in a particular organization, but also whether the intervention will be applied across a number of organizations. This question also determines the potential impact of an intervention, because organizational efforts are directed at large-scale change, whereas bottom-up execution requires educating each individual to adopt the intervention strategy (Hornung et al., 2010). However, a review of the impact of interventions on the flow-related concept of work engagement found that bottom-up interventions were more effective than top-down ones (Donaldson et al., 2019). The authors argue that this may be due to a greater likelihood of implementation errors in top-down approaches and a lack of individual autonomy. Nevertheless, bottom-up execution may be limited in its effect because individuals can only change their immediate environment (Donaldson et al., 2019). Since this limitation exists even if each person were to target her group, a combination of both bottom-up and top-down initiatives may be most effective in inducing change (Hornung et al., 2010).

**Table 1:** Guiding questions for developing flow interventions.

	Guiding questions	Options	Important considerations
1^st^ dimension	What does the intervention aim for?	Entering, Boosting, Maintaining	Current flow state
2^nd^ dimension	What does the intervention target?	Context, Individual, Group	Fix variables; Organizational restrictions
3^rd^ dimension	Who executes the intervention?	Top-down, Bottom-up	Scope of impact; Comprehensiveness versus organizational specificity

In addition to providing these guiding questions for intervention design decisions, our framework also demonstrates a concrete research agenda for future flow intervention studies. Because it would not be helpful to design interventions to maintain flow if a person is not experiencing flow at all in their current job, our framework highlights the importance of first establishing the antecedents. In line with the initial empirical studies of flow, which assumed that flow could only be experienced if the antecedent of a skill-demand-balance was present (Csikszentmihalyi and LeFevre, 1989), we argue for a thorough evaluation of the state of the antecedents before implementing an intervention. This is particularly important to avoid misleading interpretations of intervention effectiveness evaluations. For example, the lack of a significant effect of an intervention aimed at the attributes of flow (i.e., aiming for maintaining flow) could be due to the actual ineffectiveness of the intervention, but also to the absence of one of the three flow antecedents. Hence, researchers should not only design flow interventions that first aim to establish the antecedents, but then also carefully ensure that all antecedents are consistently met when evaluating interventions to boost or maintain flow.

Finally, our framework sheds light on when interventions can increase the duration, intensity, or frequency of flow. This provides researchers with guidance on which flow operationalization to use when evaluating the effectiveness of an intervention. As noted above, we adopt flow as a yes-or-no continuous phenomenon (Peifer and Engeser, 2021), such that the presence of the antecedents determines whether flow can occur at all. Thus, interventions that attempt to establish these antecedents may influence the frequency and total duration of flow experiences. It is important to note that earlier empirical studies have often adopted an exclusively continuous flow operationalization to evaluate flow interventions and then analyzed increases in flow intensity (e.g., Weintraub et al., 2021). However, this does not allow conclusions about whether an intervention supports entry into flow in the first place (i.e., crossing the threshold from nonflow to flow). Therefore, evaluations of the effectiveness of interventions that target the antecedents of flow should rather use a discrete flow measure (e.g., the Flow Questionnaire; Csikszentmihalyi and Csikszentmihalyi, 1988) to make inferences about the transition from nonflow to flow states. In contrast, interventions that modulate the moderators should be evaluated using a combination of continuous and discrete flow measures. Specifically, they should apply continuous flow measures only when a discrete flow measure indicates a flow state (Peifer and Engeser, 2021). This makes it possible to assess whether the moderator-directed intervention strengthens the relationship between the presence of the antecedents and the occurrence of flow, either by increasing the likelihood of transgression to flow, i.e., the frequency of flow experiences (assuming the antecedents are met), or by increasing the intensity (assuming the person actually experiences flow). For example, Engeser and Rheinberg (2008) applied the continuous Flow Short Scale to show that skill-demand balance leads to high flow intensity when the task is perceived as important. Applying an additional categorical flow measure could provide further insight into whether increasing task importance is helpful in facilitating the emergence of flow from a skill-demand balance. As we have argued, interventions that occur when a person is currently in flow cannot affect the total frequency of flow experiences (i.e., how often the person enters flow), but rather modulate the intensity and the duration of the current flow experience. Thus, we have argued that these interventions aim for maintaining flow. Although continuous measures alone can capture changes in flow intensity (Abuhamdeh, 2020), researchers should also assess the effectiveness of these interventions using the combination of continuous and discrete flow measures discussed above. For example, Collins et al. (2009) assessed flow intensity only on days when participants reported the presence of flow. Hence, our proposed dimension of the intervention aim directly corresponds to the person’s current flow state and, together, provides the guiding principle for how changes in flow due to an intervention should be assessed.

### 4.2. An exemplary application of the framework in research

To substantiate our aforementioned theoretical contributions with concrete guidance for the scientific field, we would like to provide an example. Imagine a researcher who decides to investigate how reducing interruptions at work fosters flow. This approach directly relates to the flow characteristic of high concentration. Hence, we can infer from our framework that the aim is to maintain flow. This aim presumes that the antecedents are fulfilled. Therefore, we recommend testing this assumption first. To do this, the researcher should conduct a pilot study that examines the presence of the antecedents in the particular setting. If a pilot study is not feasible, the researcher should at least include a control questionnaire that asks about the status of the antecedents. Next, the researcher considers the actual intervention strategy in terms of its target. A straightforward intervention to reduce interruptions for focused immersion in a task would be to target the environment by providing isolated workstations. Suppose, however, that given spatial allocations limit these changes. So the researcher decides to target the employees instead. To do so, they design a tool that helps schedule tasks depending on when the office is least busy. Lastly, the researcher considers the third dimension, the executor, which directly relates to the potential scope of the application. Since they want to evaluate the effectiveness of their tool across organizations, they decide to recruit teams from different organizations and ask the management to provide the tool for the employees as a top-down intervention. Lastly, to evaluate the intervention’s effect on flow, the researcher can follow from the aim of maintaining flow that they should use a combination of a discrete and a continuous measure, e.g., a combination of the Flow Questionnaire with the Flow Short Scale as proposed by Peifer and Engeser (2021). As you can see from this example, following the three guiding questions (Table 1) points the researcher to necessary considerations and equips them with concrete instructions for meeting constraints and evaluating the effectiveness of their study.

### 4.3. Practical implications

In addition to the theoretical contributions and implications for researchers, our framework also bears implications for practitioners. As proposed for flow researchers, organizations that want to increase the flow experiences of their employees should first and foremost strive to meet the three flow antecedents. If these antecedents are not met, efforts to increase flow will always fall short. In addition to initiating contextual changes (e.g., assigning different tasks), managers should target the individual person, for example, by providing autonomy in task choice, offering opportunities for self-learning, or allowing employees to set individual goals. Targeting the group rather than the individual may be especially promising for organizational efforts, as it allows influencing more than one person at a time. Especially with regard to today’s common collaboration in virtual teams, increasing social flow, for example by strengthening collective goal commitment or trust between group members, is important to enhance performance (Aubé et al., 2014; Breuer et al., 2016). In addition to group targeting as an effective top-down approach, this approach can also be part of bottom-up initiatives. When individuals themselves apply group-targeted strategies, they not only foster their own flow, but this effect is also transmitted to their team members. In this way, a bottom-up, group-targeted intervention becomes a time- and cost-efficient tool for enabling change on a larger scale. Generally, our framework highlights that individuals can self-initially build up their flow experiences. Hence, managers should empower their employees to take responsibility for their flow by educating them about the beneficial effects of flow and potential flow-fostering strategies. Importantly though, this does not absolve organizations from their responsibility to creating the necessary foundations for flow to arise.

As mentioned before, “individuals greatly differ in the *need* to seek and in the *ability* to create flow experiences” (Baumann, 2021, p. 251). This can lead to frustrated reactions to flow interventions (e.g., reward systems based on flow experiences) by persons high *and* low in flow proneness. Employees who do not experience flow easily may feel discriminated against by these reward systems. Therefore, employment protection policies need to establish guidelines for recognizing individual baselines. That said, the use of extrinsic rewards for flow may also negatively affect individuals who self-initiate tasks that allow them to experience flow. Since these individuals are already intrinsically motivated, the extrinsic reward could undermine their motivation (Deci et al., 1999). Thus, practitioners should always begin with an analysis of the status quo of flow experiences in their target group. As part of this initial assessment, they should also analyze whether flow is mostly experienced in solitary or interactive tasks. This will help determine if and in what situations individuals are already experiencing high flow. If they experience flow only when working alone, it may be promising to address group-related targets, such as assigning interdependent tasks, for fostering social flow. In contrast, if there is high interindividual variability in flow, it would be more appropriate to use an individualized approach that targets each person directly.

Since flow at work not only improves performance, but also increases individual job satisfaction and general well-being (Peifer and Wolters, 2021), it is of great societal interest to foster flow across work domains. To this end, our framework also provides a starting point for training initiatives in education that go beyond educating managers to empower their employees. For example, by applying strengths use interventions and promoting self-regulation skills in adolescents, schools can already help students with choosing work domains that allow them to experience flow more often.

### 4.4. Limitations and avenues for future research

As with any model, our framework entails certain limitations. First, we aimed to generate a framework with strong heuristic value, thus minimizing the number of dimensions for classification. However, this meant neglecting other potential dimensions, such as the targeted timeframe. In order to provide flow-specific guidance, we also omitted classifications related to general intervention format, such as type of delivery (e.g., web-based, paper-based). Although we recognize the resulting loss of an all-encompassing classification, we strongly advocate that future research first focus on developing flow interventions with careful consideration of content. Only then should they investigate whether the effectiveness of an intervention changes due to modulations in format. By that, the effectiveness of a specific intervention can be validated without confounding it with format-related influences.

A second limitation of our framework emerges from the theoretical overlap between flow and other work-related concepts (e.g., work engagement). Because of this overlap, future empirical research may find that strategies for fostering these concepts are largely similar to those for flow. However, since flow is not synonymous with these concepts, especially in terms of its conceptualization as an optimal state, we do not assume that any intervention for similar concepts could induce this particular experience. Nevertheless, we suggest evaluating the influence of a flow intervention on closely related concepts as well. Since organizations cannot implement an infinite number of interventions due to limitations in resources and time, strategies that simultaneously affect more than one desirable outcome are especially likely to be applied in the workplace.

Third, the proposition to implement flow interventions from the bottom-up could be interpreted as implying that the person is responsible for not experiencing flow. They would then be to blame for missing out on the benefits associated with flow. This assumption is one of the most harmful interpretations of strategies that stress the importance of individual agency because it completely ignores the causal strength of contextual factors (e.g., socioeconomic status). Their constitution can impede a person’s well-being regardless of how much effort that person puts into improving their state. Thus, the categories of our framework’s dimensions should not be interpreted as a range of options from which researchers or practitioners should choose only one. Instead, strategies for fostering flow can only help if they are part of a comprehensive approach that targets each side of the coin.

Fourth, successfully fostering flow is not necessarily a morally good thing, especially if flow is experienced in unethical activities (Zimanyi and Schüler, 2021). Independent of the specific task, flow does not have only beneficial effects, but also bears certain dangers (for a full discussion of potential harms, see Zimanyi and Schüler, 2021). For example, in order to experience a balance between skills and demands at work, a person needs to tackle challenging tasks. This increases the likelihood of making mistakes because failure to achieve this balance can result in a state of excessive demand. Also, although flow feels effortless (Moller et al., 2013), it is an energy-consuming state that can lead to severe exhaustion (Zimanyi and Schüler, 2021). In particular, if an organization strongly promotes flow-fostering interventions, this will probably exert pressure on employees because it implies that they should be in flow all the time. However, since flow is an optimal state, this is neither likely nor desirable. Also, since flow is an intrinsically motivating state, experiencing it in certain tasks may incline a person to neglect other tasks. Thereby, flow can resemble and be conducive to addiction (Zimanyi and Schüler, 2021). Hence, organizations and individuals should refrain from concluding that flow should be fostered at all costs, but rather carefully evaluate when and why flow experiences are desirable.

Lastly, flow is a highly fluctuating state with significant individual and situational variability (Fullagar and Kelloway, 2009; Ceja and Navarro, 2011). Hence, an overarching framework for systematizing flow interventions may neglect the fact that flow-fostering strategies need to be adaptive to the individual and the situation. This does not only relate to the already discussed differences in general flow proneness. For example, although flow at work is associated with higher energy levels in leisure time (a strong individual benefit), this depends on whether the person succeeds at psychologically detaching from work at home (Demerouti et al., 2012). Similarly, whether a person has a harmonious or obsessive passion for a task (Vallerand, 2015) determines the relationship of that task with experiencing flow, as well as detrimental effects on experiencing flow in other tasks (Carpentier et al., 2012). As discussed above, interventions that apply when a person is currently experiencing flow, may even immediately disrupt the experience. Hence, it is not only ineffective, but potentially harmful to apply similar interventions across individuals and situations. We thus encourage future research to develop adaptive flow interventions that, for example, only apply when a person is not currently in flow. The detection of (neuro-)physiological correlates of flow (for a review see Peifer and Tan, 2021) as objective and high-frequency flow markers for real-time measurements is a promising starting point to enable these adaptive mechanisms.

## 5. Conclusion

In sum, we have proposed a three-dimensional framework with strong heuristic value that allows the systematization of flow interventions according to their specific aim, target, and executor. We advocate that future research should first develop interventions to establish the antecedents of flow before moving on to the inherent attributes of flow experiences. By acknowledging the individual and situational variability of flow, we emphasize the importance of developing adaptive mechanisms in the application of interventions. While being in flow all the time cannot and should not be the ultimate goal, we believe that these adaptive flow interventions will ultimately increase organizational performance and help individuals thrive at work.

## Author contributions

KB conceived of the presented idea and developed the framework. KB and MK wrote the manuscript. CW supervised the writing process. All authors discussed the article and contributed to the final manuscript.

## Funding

Funded by the Deutsche Forschungsgemeinschaft (DFG, German Research Foundation)—GRK2739/1—Project Nr. 447089431—Research Training Group: KD^2^School—Designing Adaptive Systems for Economic Decisions.

## Conflict of interest

The authors declare that the research was conducted in the absence of any commercial or financial relationships that could be construed as a potential conflict of interest.

## Publisher’s note

All claims expressed in this article are solely those of the authors and do not necessarily represent those of their affiliated organizations, or those of the publisher, the editors and the reviewers. Any product that may be evaluated in this article, or claim that may be made by its manufacturer, is not guaranteed or endorsed by the publisher.
